# Autophagy-Targeting Stapled Peptide Utilizes Macropinocytosis for Cell Entry to Potentiate Anti-Proliferative Autosis in Small-Cell Lung Cancer

**DOI:** 10.3390/pharmaceutics17121521

**Published:** 2025-11-26

**Authors:** Jingyi Chen, Shan Gao, Xiaozhe Zhang, Na Li, Yingting Yu, Lei Wang, Yu Feng, Yuanzhi Lao, Yanxiang Zhao

**Affiliations:** 1 Department of Applied Biology and Chemical Technology, The Hong Kong Polytechnic University, Hung Hom, Kowloon, Hong Kong 999077, China; jing2chen@polyu.edu.hk (J.C.); gspolyu@gmail.com (S.G.); xiaozhe2.zhang@polyu.edu.hk (X.Z.); ying-ting.yu@polyu.edu.hk (Y.Y.); 20034105R@connect.polyu.hk (L.W.); fengyu.feng@polyu.edu.hk (Y.F.); 2 Shenzhen Research Institute, The Hong Kong Polytechnic University, Shenzhen 518057, China; na.li.li@connect.polyu.hk; 3 Collaborative Innovation Department, Simcere Pharmaceuticals Group, Nanjing 210042, China; laoyuanzhi@simcere.com

**Keywords:** small cell lung cancer, autophagy, macropinocytosis, autosis

## Abstract

**Background**: Small-cell lung cancer (SCLC) is an aggressive malignancy marked by rapid progression, early metastasis, and frequent relapse despite chemotherapy. Due to its genetic complexity, targeted therapies have limited success. Autophagy, a lysosome-dependent cellular degradation process, plays a key role in SCLC, yet effective autophagy-targeting strategies are lacking. This study evaluates Tat-SP4, an autophagy-targeting stapled peptide, for its anti-proliferative effects in SCLC. **Method**: We assessed Tat-SP4′s impact on autophagy in SCLC cells by measuring p62 and LC3 levels. Mitochondrial function was evaluated via mitochondrial membrane potential (Δψm) and oxygen consumption rate (OCR). Anti-proliferative effects were determined using cell viability assays in vitro and xenograft models in vivo. Cellular uptake mechanisms were investigated using Ca^2+^ imaging and pharmacological inhibitors. **Result**: Tat-SP4 induced a strong autophagic response and triggered autosis, a form of autophagy-dependent necrotic cell death, impairing SCLC cell proliferation. It also caused mitochondrial dysfunction with impaired oxidative phosphorylation (OXPHOS). Tat-SP4 entered cells predominantly via macropinocytosis, triggering extracellular Ca^2+^ influx measurable by live-cell imaging. Digoxin, an Na^+^, K^+^-ATPase inhibitor, partially reversed the effect of Tat-SP4 on Ca^2+^ influx, cell death, and OXPHOS activity. Lastly, Tat-SP4 inhibited tumor growth in a xenograft-based animal model for SCLC. **Conclusions**: The autophagy-targeting stapled peptide Tat-SP4 inhibited the proliferation of SCLC cells in vitro and inhibited the growth of the SCLC tumor in vivo. Macropinocytosis facilitates cell entry for Tat-SP4, which can be monitored by influx of extracellular Ca^2+^. By exploiting macropinocytosis for cell entry and converting the pro-survival autophagy process into a death pathway, Tat-SP4 represents a novel therapeutic strategy against SCLC.

## 1. Introduction

Small-cell lung cancer (SCLC) is a highly aggressive neuroendocrine malignancy that accounts for 10–15% of all lung cancer cases worldwide. SCLC is strongly linked to tobacco smoking, with over 95% of cases occurring in current or former smokers [1]. SCLC has a poor prognosis, with a median survival of only 9–12 months for cases diagnosed at the extensive stage (ES) with distant metastases. Even cases diagnosed at the limited stage (LS), with the tumor confined to just one hemithorax, still have a poor median survival of only 15–20 months [2,3].

Platinum-based chemotherapy, typically cisplatin or carboplatin combined with etoposide, has served as first-line treatment for both LS- and ES-SCLC for decades [4]. Although most SCLC patients respond well to chemo- and radiotherapy initially, relapse occurs frequently and leads to poor long-term survival. New therapeutic approaches, particularly the combination of chemotherapy with immune checkpoint inhibitors targeting the PD-L1/PD-1 axis, have been approved in recent years as second-line treatment for SCLC. However, these combination therapies have a low overall response rate of 10–20% and a limited overall survival benefit of 2–3 months [5,6]. Other combination strategies, such as chemotherapy with targeted agents, including PARP inhibitors [7] and DLL3-directed antibody-drug conjugates [8], failed to demonstrate durable efficacy in late-phase clinical trials.

Autophagy is a conserved lysosomal degradation process and plays a dual-natured context-dependent role in cancer [9]. In the early stage of tumorigenesis, such as LS-SCLC, autophagy is regarded as playing an anti-tumor role by maintaining cellular homeostasis and suppressing malignant transformation. In the late stage of cancer disease, such as ES-SCLC, autophagy likely plays a pro-tumor role by sustaining the proliferation of cancer cells under metabolic and therapeutic stress [9,10,11]. Both autophagy inhibitors like chloroquine, and enhancers, like rapamycin, have shown anti-proliferative effects in multiple cancer types, including SCLC [12]. However, these modulators are not autophagy-specific, and the feasibility of targeting autophagy to inhibit SCLC tumor growth requires further investigation.

In our previous studies, we developed stapled peptides that targeted Beclin 1, a key autophagy protein and a tumor suppressor with frequent monoallelic deletion in several cancer types [13]. One lead candidate, Tat-SP4, showed strong binding to Beclin 1 with micromolar affinity and triggered an autophagic response in vitro [14]. Tat-SP4 also exerted a potent anti-proliferative effect in several cancer cell lines, including triple-negative breast cancer (TNBC) [15], pancreatic ductal adenocarcinoma (PDAC) [16], hepatocellular carcinoma (HCC) [17], and non-small-cell lung cancer (NSCLC) [18]. Importantly, scrambled control peptides, such as Tat-SC4—which retains the same N-terminal Tat sequence but incorporates a randomized C-terminal segment that does not bind to Beclin 1—have been rigorously validated in previous studies and shown to lack Beclin 1-binding activity as well as any autophagy-inducing or anti-proliferative effects [14,15,16,19]. These prior results established the specificity of Tat-SP4 and provide the rationale for using PBS or no treatment as the baseline control in the current work. In this study, we investigate whether Tat-SP4 would exert a similar anti-proliferative effect in SCLC. We also studied how Tat-SP4 gained cell entry to achieve such an effect, an important but often understudied aspect for peptide-based therapeutics. Our results reveal that Tat-SP4 utilized macropinocytosis for cell entry and triggered autosis, a form of autophagy-dependent cell death, in SCLC cells to inhibit tumor growth. These results validate autophagy-targeting stapled peptides as a potential therapeutic modality for SCLC and highlight the importance of macropinocytosis to facilitate this approach.

## 2. Materials and Methods

### 2.1. Cell Lines and Cell Culture

The small cell lung cancer cell lines, SHP-77 (RRID: CVCL_1693) and H146 (RRID: CVCL_1473), were obtained from American Type Culture Collection (ATCC). They were cultured in Roswell Park Memorial Institute (RPMI) 1640 Medium (Gibco, Waltham, MA, USA) with the supplementation of 10% fetal bovine serum (FBS, Gibco). Cells were cultured in 5% CO_2_, 37 °C incubators. All cell lines used in the experiments were mycoplasma-detected negative by MycoAlert™ PLUS Mycoplasma Detection Kit (Lonza, Walkersville, MD, USA) before and during the experiment.

### 2.2. Reagents and Antibodies

Chloroquine (CQ; Sigma-Aldrich, St. Louis, MO, USA), Epidermal Growth Factor (EGF; Gibco), EDTA-free protease inhibitor cocktail (Roche, Basel, Switzerland), Trypsin (Invitrogen, Waltham, MA, USA), Trypan Blue (Gibco), Z-VAD-FMK (Sigma-Aldrich), Digoxin (MedChemExpress, Monmouth Junction, NJ, USA), Ferristatin-1 (Fer-1; Sigma-Aldrich), Erastin (Sigma-Aldrich), Necrostatin-1 (Nec-1; Sigma-Aldrich), Oligomycin (Cayman, Ann Arbor, MI, USA), FCCP (Cayman), Rotenone (Sigma-Aldrich), Antimycin A (Sigma-Aldrich), Matrigel (Corning, Corning, NY, USA), Heparin (MedChemExpress), Amiloride (MedChemExpress), Methyl-beta-cyclodextrin (MβCD, MedChemExpress), anti-β-actin antibody (Santa Cruz Biotechnology, Santa Cruz, CA, USA; sc-47778; RRID:AB_626632;1:2500 dilution), anti-LC3 antibody (Novus, Centennial, CO, USA; NB100-2220; RRID:AB_10003146; 1:1000 dilution), anti-p62 antibody (Abnova, Taipei, Taiwan; H00008878-M01; RRID:AB_548364; 1:10,000 dilution), Anti-Mouse IgG-HRP (Sigma-Aldrich; A9044; RRID:AB_258431; 1:2500 dilution), Anti-Rabbit IgG-HRP (Sigma-Aldrich; A9169; RRID:AB_258434; 1:2500 dilution)

### 2.3. Cell Viability Assays

Cell viability was measured by a trypan blue exclusion assay following the standard protocol provided by the manufacturer. SHP-77 cells and H146 cells were plated into 96-well plates at a density of 2 × 10^4^ cells/well. Upon cell attachment, cells were treated with the graded concentrations of Tat-SP4 for 24 h. The IC_50_ value was calculated from the curve fitted to concentration–response data sets. All experiments were repeated in triplicate, and the mean was calculated. For the 5-day growth curve, cells were seeded into a 24-well plate at 2.5  ×  10^4^ cells/well for SHP-77 and H146 cells. Cells were treated with 10 μM Tat-SP4. Cells were allowed to grow for up to 5 days post-treatment, and the viable cell number was checked every 24 hours with the trypan blue exclusion assay. For cell death rescue assays, SHP-77 cells were first plated overnight in 96-well plates. Tat-SP4 was added to the cells, together with inhibitors for apoptosis, ferroptosis, necroptosis, or autosis. After 24 h treatment, the cell viability was measured by the trypan blue exclusion assay.

### 2.4. Immunoblot Analysis

Cells were lysed in Laemmli sample buffer containing 62.5 mM Tris-HCl (pH 6.8), 2% SDS, 25% glycerol, and 5% β-mercaptoethanol, along with an EDTA-free protease inhibitor cocktail. Protein concentrations were measured using the Bio-Rad Protein Assay (Bio-Rad, Hercules, CA, USA). Proteins were then separated by SDS-PAGE and transferred onto a PVDF membrane (Millipore, Darmstadt, Germany). The membrane was incubated with primary antibodies overnight at 4 °C to detect the target proteins, followed by a 1 h incubation with secondary antibodies at room temperature. Protein bands were visualized using ECL reagents, with β-actin used as a loading control.

### 2.5. Flow Cytometry

Cellular apoptosis and necrosis were assessed by flow cytometry following standard protocols. Briefly, SHP-77 cells were harvested by trypsin after the indicated peptide treatment, labeled with Annexin V and PI (Invitrogen), loaded into the flow cytometer system (BD Accuri C6), and analyzed by BD Accuri C6 software (version 1.0.264.21). The cell populations were identified by Annexin V/PI staining. Typically, the cellular population in the lower left quadrant represented live cells (Annexin V^−^, PI^−^), the lower right quadrant represented early apoptotic cells (Annexin V^+^, PI^−^), the upper right quadrant represented late apoptotic cells (Annexin V^+^, PI^+^), and the upper left quadrant represented necrotic cells (Annexin V^−^, PI^+^). The change in mitochondrial membrane potential (∆ψm) was assessed by measuring the fluorescence intensity of tetramethylrhodamine methylester (TMRM). Cells cultured in 6-well plates were treated with Tat-SP4. After treatment, the culture medium was removed, and the cells were stained with 100 nM TMRM, followed by an additional 30 min incubation at 37 °C. After incubation, cells were harvested by trypsinization and analyzed using a flow cytometer to detect fluorescence emission at 570 nm. Reactive oxygen species (ROS) production was measured using the fluorescent intensity of the CellROX green probe (Thermo Fisher Scientific, Waltham, MA, USA). Cells were treated with Tat-SP4, followed by trypsinization. The collected cells were stained with CellROX reagent at a final concentration of 500 nM and incubated for 1 h at 37 °C. After incubation, the cells were washed three times with cold PBS. ROS generation was detected using flow cytometry, with fluorescence emission measured at 530 nm. For each sample, at least 20,000 events were recorded and analyzed using BD Accuri C6 software.

### 2.6. Seahorse Analysis

Oxygen consumption in intact cells was assessed using an XFe24 Extracellular Flux Analyzer (Agilent, Santa Clara, CA, USA) following the manufacturer’s standard protocol. Briefly, one day prior to the experiment, an XF24 cell culture microplate was coated with 100 µg/mL poly-lysine and incubated in a CO_2_ incubator at 37 °C. On the same day, 1 mL of XF calibrant was added to each well of the utility plate to hydrate the sensor, and the XF24 sensor cartridge was incubated overnight in a non-CO_2_ incubator at 37 °C. On the day of the experiment, the coated XF24 cell culture microplate was washed with PBS and then allowed to air dry. Then, SHP-77 cells were seeded at a density of 1.5 × 10^5^ cells per well in the coated XF24 cell culture microplate with the XF assay medium containing 1 mM sodium pyruvate, 2 mM glutamine, and 10 mM glucose and incubated for 1 h in a non-CO_2_ incubator at 37 °C.

### 2.7. Intracellular Ca^2+^ Measurement

Genetically encoded Ca^2+^ indicators used in this study include mito-GCaMP5G, cyto-RCaMP1h, and G-CEPIA1er. SHP-77 cells were plated in 6-well plates to allow transfection. Cells were transfected with either mito-GCaMP5G, cyto-RCaMP1h, or G-CEPIA1er cDNA using Lipofectamine 2000 and incubated for 36 h. After transfection, cells were collected and evenly distributed into 24-well plates in HBSS buffer (Gibco) for imaging. Time-lapse fluorescence imaging was performed using an Olympus inverted epifluorescence microscope equipped with a ×20 objective and appropriate excitation/emission filter sets. Images were acquired every 3 s.

To monitor subcellular Ca^2+^ dynamics, SHP-77 cells expressing mito-GCaMP5G, cyto-RCaMP1h, or G-CEPIA1er were imaged to capture Ca^2+^ fluctuations in the mitochondria, cytosol, and endoplasmic reticulum, respectively. Tat-SP4 or other agonists were applied 60 s after the start of imaging. Fluorescence signals were quantified as ΔF/F_0_, where ΔF represents the change in fluorescence at each time point and F_0_ denotes the baseline fluorescence.

### 2.8. Animal Study

Animal experiments were carried out according to the guidelines of the Centralized Animal Facilities of the Hong Kong Polytechnic University. Male nude mice, 5–6 weeks in age, were purchased from the Centralized Animal Facilities of the Hong Kong Polytechnic University. Exponentially growing SHP-77 cells were harvested and resuspended in PBS with a concentration of 1 ×10^8^ cells per ml. Mixed cell suspensions with Matrigel (Corning) in a 1:1 volume ratio with 5 × 10^5^ SHP-77 cells were injected subcutaneously into the right-lower back of the mice. The tumor volume was calculated by the equation of Length × Width × Width/2. Mice were divided randomly into two groups (8 animals/group) when the tumor volume reached 50–60 mm^3^. Tat-SP4 was administered intraperitoneally at 40 mg/kg every day, while PBS was administered to mice in the control group. The tumor volume was measured every other day. Treatments were continued until the tumor volume in the mice in the control group reached the maximum volume allowable according to the Centralized Animal Facilities of the Hong Kong Polytechnic University. At the end of the experiment, mice were sacrificed, and both tumors and vital organs were harvested for analysis. Tumor weights were measured after resection.

## 3. Results

### 3.1. Beclin 1-Targeting Stapled Peptide Tat-SP4 Promotes Autophagy in SCLC Cells

**Figure 1:**
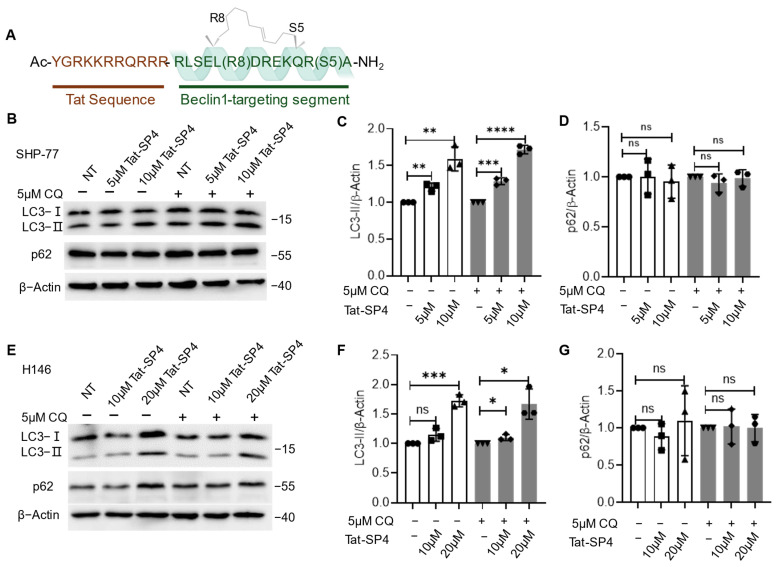
Tat-SP4-induced autophagy in SCLC cell lines. (**A**) Tat-SP4 consists of a cell-penetrating Tat sequence (highlighted in brown) at the N-terminus and a Beclin-1-targeting segment at the C-terminus. The α-helical conformation is stabilized by a hydrocarbon staple. (**B**) Western blot to assess the p62 level and LC3 lipidation profile in SHP-77 cells after treatment with 5μM and 10 μM of Tat-SP4 for 3 h, in the presence or absence of CQ. (**C**) Quantification of LC3 lipidation profiles. (**D**) Quantification of p62 levels from the Western blot data. (**E**) Similar Western blots to (**B**), but cells are H146. (**F**,**G**) Quantification of LC3 and p62 levels from data in (**E**). The levels of LC3-II or p62 were normalized to the β-Actin level. Data are presented as mean ± SEM (*n* = 3). * *p* < 0.05, ** *p* < 0.01, *** *p* < 0.001, **** *p* < 0.0001, unpaired *t*-test.

Our autophagy-targeting stapled peptides were designed to contain an N-terminal Tat sequence for cell penetration and a C-terminal segment for specific binding to the coiled coil domain of Beclin 1 (Figure 1A) [14]. Our previous studies showed that one lead candidate, Tat-SP4, induced autophagy and inhibited cell proliferation in several cancer types, including TNBC [15], ovarian cancer [19], liver cancer [17], and NSCLC [18]. Encouraged by these findings, we decided to investigate whether Tat-SP4 would exert a similar effect in two genetically and phenotypically distinct SCLC cell lines, SHP-77 and H146. Specifically, SHP-77 is of the variant SCLC type with less neuroendocrine features and a high level of autophagy, while H146 is of the classical SCLC type with mutations in tumor suppressors p53 and RB1 and reduced autophagy flux [20]. Such heterogeneity may help to assess how SCLC cells respond to autophagy modulators like Tat-SP4 in a context-dependent manner.

The impact of Tat-SP4 on cellular autophagy was assessed by tracking the autophagy markers LC3 and p62. Our results show that Tat-SP4 induced a robust autophagic response in both SHP-77 (Figure 1B–D) and H146 (Figure 1E–G) cells. In SHP-77 cells, Tat-SP4 significantly increased LC3-II levels in a dose-dependent manner, and this effect was further enhanced when combined with chloroquine (CQ), a lysosomal inhibitor (Figure 1B,C). However, p62 levels remained largely unchanged (Figure 1B,D). Similar results were observed in H146 cells, with a significant increase in LC3-II level after Tat-SP4 treatment but no significant change in p62 level, whether in the presence or absence of CQ (Figure 1E–G). It is well accepted that the change in LC3-II level is a more sensitive signal for autophagy activity, while p62 can be influenced by other factors [21,22]. However, the control peptide Tat-SC4 exerted no significant effect on LC3-II or p62 levels in either SHP-77 or H146 cells. (Supplementary Figure S1A–F). Overall, our data confirms that Tat-SP4 enhanced autophagy activity in SCLC cells regardless of their genetic background.

### 3.2. Tat-SP4 Inhibits Proliferation of SCLC Cells In Vitro

**Figure 2:**
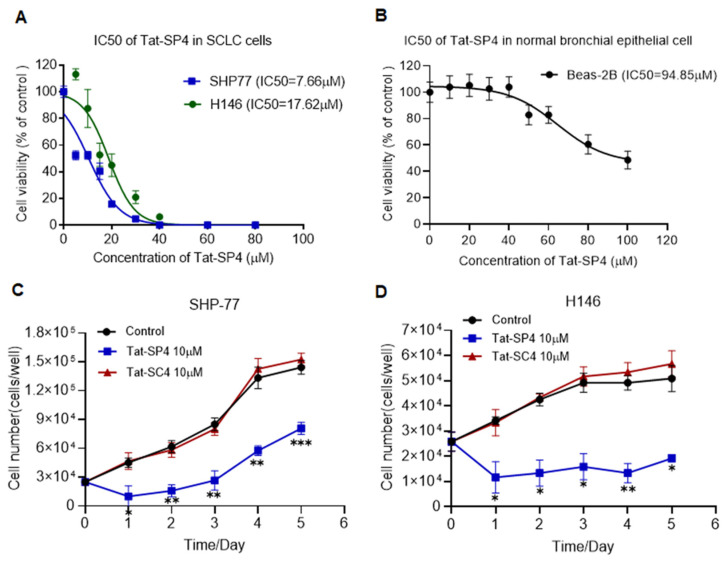
Anti-proliferative efficacy of Tat-SP4 in SCLC cell lines. (**A**) Trypan blue exclusion assay to assess the cytotoxicity IC50 of Tat-SP4 in SHP-77 and H146 cell lines. Cells were treated with Tat-SP4 at a series of concentrations for 24 h. The number of viable cells was determined manually using a hemocytometer and the trypan blue dye exclusion method. (**B**) Cytotoxicity of Tat-SP4 in normal bronchial epithelial cells by trypan blue exclusion assay. (**C**,**D**) 5-day cell proliferation curve of SHP-77 and H146 cells with 10 μM Tat-SP4 or Tat-SC4 treatment. The treatments were initiated on day 0, and cell numbers were measured every 24 h. Data are presented as mean  ±  SEM from three independent experiments; * *p* < 0.05, ** *p* < 0.01, *** *p* < 0.001, unpaired *t*-test.

Similar to our previous studies, we used trypan blue exclusion assays to assess whether Tat-SP4 would inhibit the proliferation of SHP-77 and H146 cells. Our data showed that Tat-SP4 showed a moderate anti-proliferative effect in both cell lines, with IC_50_ values of 7.66 μM and 17.62 μM, respectively (Figure 2A). In contrast, the control peptide Tat-SC4 did not induce any significant cytotoxicity in either SHP-77 or H146 cells (Supplementary Figure S2). In comparison, Tat-SP4 showed almost no anti-proliferative effect in non-cancerous bronchial epithelial Beas-2B cells, with an IC_50_ of 94.85 μM (Figure 2B), which indicates selective cytotoxicity toward malignant cells. Consistent with these findings, a 5-day proliferation assay revealed that 10 μM Tat-SP4 attenuated SHP-77 cell proliferation by approximately 50% (Figure 2C) and completely suppressed H146 cell proliferation (Figure 2D). In comparison, no anti-proliferative effect was observed in either cell line for Tat-SC4 (Figure 2C,D).

### 3.3. Tat-SP4 Induces Autotic Cell Death in SCLC Cells

**Figure 3:**
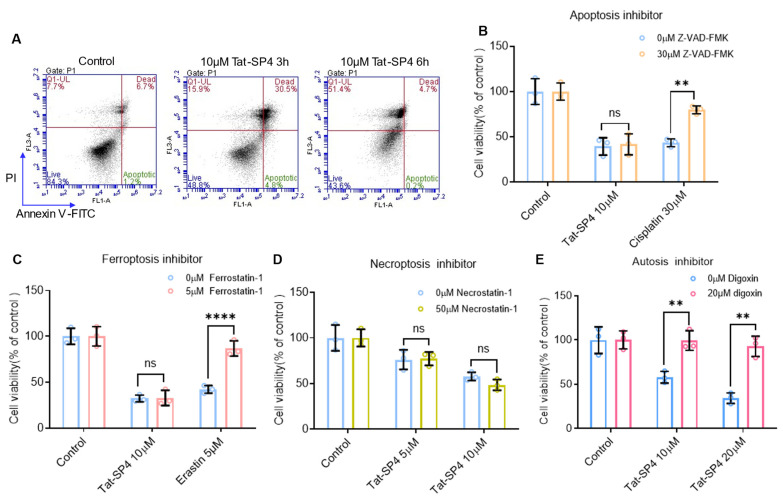
Cell death induced by Tat-SP4 is rescued by an inhibitor of autosis but not by inhibitors for other cell death programs. (**A**) Flow cytometry analysis using Annexin V FITC-PI staining was conducted on SHP-77 cells after indicated hours of treatment with the Tat-SP4. The percentage of dead cells (PI positive) in the Tat-SP4 treatment groups showed a significant increase compared to the control group, with no notable changes observed in the population of apoptotic cells. (**B**) In the absence or presence of Z-VAD-FMK, SHP-77 cells treated with Tat-SP4 or cisplatin for 24 h. (**C**) SHP-77 cells were pretreated with ferroptosis inhibitor, ferrostatin-1, followed by 10 μM Tat-SP4 or 5 μM Erastin treatment for 24 h. Ferrostatin-1 did not rescue SHP-77 cell death caused by Tat-SP4. (**D**) SHP-77 cells were treated with Tat-SP4 at 10 μM for 24 h, either in the presence or absence of necrostatin-1, respectively. Necrostatin-1 did not rescue SHP-77 cell death caused by Tat-SP4. (**E**) SHP-77 cells were pretreated with digoxin for 2 h, followed by the treatment of Tat-SP4 for 12 h. Cell viability was measured by trypan blue exclusion assay. Data are presented as mean ± SEM (*n* = 3). ** *p* < 0.01, **** *p* < 0.0001; unpaired *t*-test.

Our previous study showed that Beclin 1-targeting stapled peptides induced autosis, which is a form of autophagy-dependent necrotic cell death, in TNBC and NSCLC cells [15,18]. We investigated whether Tat-SP4 would exert a similar effect on SCLC cells. Flow cytometry with annexin V and propidium iodide (PI) staining showed that Tat-SP4 induced necrotic cell death, but not apoptosis in SHP-77 cells (Figure 3A). Specifically, treatment with 10 μM Tat-SP4 for 3 h resulted in approximately 30% necrotic cells (positive for both Annexin V and PI) and less than 5% apoptotic cells. After 6 h, nearly 50% of cells were necrotic, with minimal apoptosis.

We further characterized the cell death induced by Tat-SP4 using inhibitor rescue assays. Our data showed that Z-VAD-FMK, a pan-caspase inhibitor, rescued apoptotic cell death induced by cisplatin but had no effect on Tat-SP4-induced necrotic cell death (Figure 3B). Similar results were also observed for ferrostatin-1, an inhibitor of ferroptosis (Figure 3C), and necrostatin-1, an inhibitor of necroptosis (Figure 3D). These findings suggest that the necrotic cell death induced by Tat-SP4 in SCLC cells is distinct from apoptosis, ferroptosis, and necroptosis.

Autosis is a distinct form of necrotic cell death mediated by autophagic machinery [23]. It is pathologically triggered by prolonged starvation, hypoxia-ischemia, or viral infections, and experimentally induced by elevated concentrations of the autophagy-inducing peptide Tat-Beclin 1 in both cellular systems and in vivo models [24]. Autosis requires functional Na^+^, K^+^-ATPase—an ion pump critical for maintaining transmembrane electrochemical gradients and signal transduction. Crucially, this pathway is inhibited by Na^+^, K^+^-ATPase antagonists like digoxin [24]. Therefore, we tested whether digoxin would rescue Tat-SP4-induced cell death in SCLC cells. Our data shows that digoxin pretreatment significantly attenuated Tat-SP4-induced cytotoxicity in SHP-77 cells (Figure 3E). This finding confirms that the cell death program induced by Tat-SP4 in SHP-77 cells is autosis, a Na^+^, K^+^-ATPase-mediated form of autophagic cell death.

### 3.4. Tat-SP4 Causes Mitochondrial Dysfunction and Impairs OXPHOS Activity

Previous work has reported that autosis induced by the Tat-Beclin 1 peptide in HeLa cells displayed ultrastructural alterations in multiple organelles preceding cell death [24]. Consistently, our prior study showed that the autosis induced by Tat-SP4 in TNBC cells was accompanied by distinctive mitochondrial damage, including cristae disruption and swelling [15]. Given the essential role of mitochondria in coordinating multiple death pathways, we assessed the impact of Tat-SP4 on mitochondrial function.

**Figure 4:**
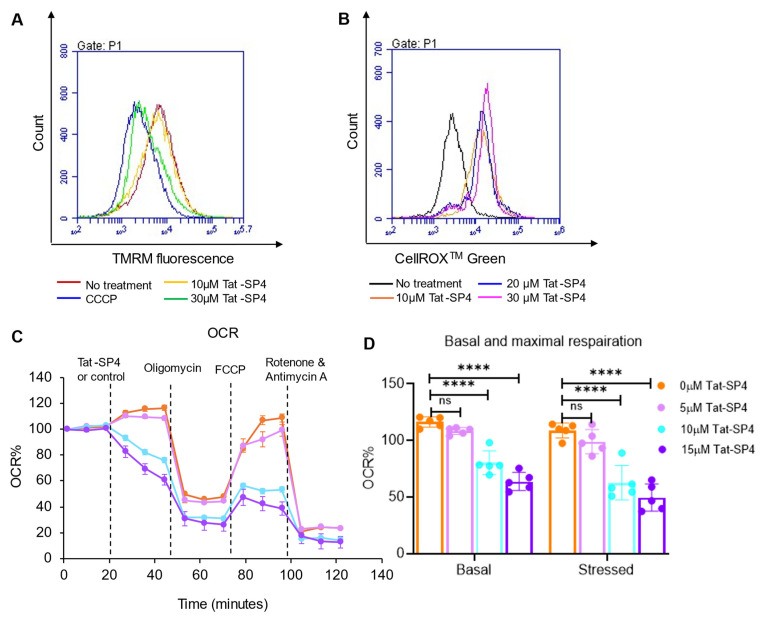
Tat-SP4 impairs mitochondrial function and OXPHOS activity. (**A**) Representative flow cytometry plot of SHP-77 cells with the treatment of indicated concentrations of Tat-SP4 for 30 min. Cells were stained with TMRM for 30 min and analyzed using flow cytometry. (**B**) Representative flow cytometry plot of SHP-77 cells treated with indicated concentrations of Tat-SP4 for an hour, followed by CellROXTM green probe staining. Fluorescence intensity is directly proportional to the amount of ROS in the cell. (**C**) OCR was measured using the Mito Stress Test with Agilent Seahorse Analyzer. SHP-77 cells were treated with either Tat-SP4 or vehicle (control), 1 μM oligomycin, 1 μM FCCP, and 0.5 μM rotenone and antimycin A sequentially. Each data point represented a real-time OCR measurement. (**D**) Quantification of basal maximum respiration changes respiration after Tat-SP4 treatment and in (**A**). **** *p* < 0.0001.

Our data showed that, in SHP-77 cells, Tat-SP4 induced a dose-dependent dissipation of mitochondrial membrane potential (ΔΨm), a key indicator for mitochondrial function (Figure 4A). Consistent with such mitochondrial depolarization, Tat-SP4 also elevated cellular ROS levels in SHP-77 cells in a dose-dependent manner (Figure 4B). As ΔΨm is essential for the function of the electron transport chain (ETC) and oxidative phosphorylation (OXPHOS), we inspected whether Tat-SP4 would disrupt this important bioenergetics process [25]. We used Agilent Seahorse XF analysis to measure the basal and maximal oxygen consumption rate (OCR), which serve as indicators of intrinsic OXPHOS activity and uncoupled ETC capacity, respectively. Our data shows that Tat-SP4 reduced the basal OCR in SHP-77 cells by approximately 30% and 40% at concentrations of 10 µM and 15 µM, respectively (Figure 4C,D). Similarly, Tat-SP4 also reduced the maximal OCR by roughly 40% and 50% at 10 µM and 15 µM, respectively (Figure 4C,D). These results confirm that Tat-SP4 causes mitochondrial dysfunction and impairs OXPHOS activity in SCLC cells.

### 3.5. The Cell Entry of Tat-SP4 Is Mediated by Macropinocytosis

Our autophagy-targeting stapled peptides including Tat-SP4 used the well-characterized Tat sequence to facilitate cell entry [14]. Cell-penetrating peptides (CPPs) like Tat are thought to promote the internalization of cargos through multiple pathways, such as direct translocation across the plasma membrane or endocytic pathways, but the mechanistic details of these pathways are not well characterized [26].

**Figure 5:**
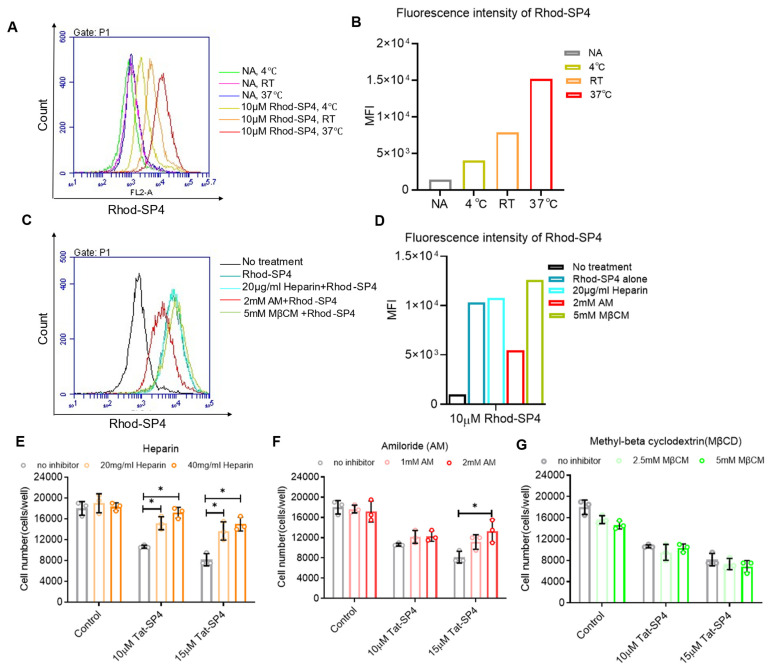
The cellular uptake process of Tat-SP4 is energy- and macropinocytosis-dependent. (**A**) Representative flow cytometry plot of SHP-77 cells stained with Rhod-SP4 at 10 μM at 4 °C, room temperature, and 37 °C for an hour, respectively. The fluorescence intensity of Rhod-SP4 increases with increasing temperature. (**B**) Mean fluorescence intensity of Rhod-SP4 from (**A**). The bar graph represents a single experiment without replicates. (**C**) Representative flow cytometry plot of SHP-77 cells stained with Rhod-SP4 at 10 μM at 37 °C for an hour, in the presence or absence of various inhibitors. AM, Amiloride; MβCD, Methyl-beta cyclodextrin. Cells were pretreated with different inhibitors for 30 min, followed by staining with Rhod-SP4 at 10 μM. (**D**) Mean fluorescence intensity of Rhod-SP4 from (**C**). The bar graph represents a single experiment without replicates. (**E**–**G**) Assess the effect of different inhibitors of cell-penetrating peptides uptake pathways on Tat-SP4-induced cell death. (**E**) Heparin, with its negative charge, rescued Tat-SP4-induced cell death significantly. (**F**) Amiloride, an inhibitor of micropinocytosis, did not rescue Tat-SP4-induced cell death. (**G**) Methyl-beta-cyclodextrin, an inhibitor of lipid raft-mediated endocytosis, did not rescue Tat-SP4-induced cell death. Data are presented as mean ± SEM (*n* = 3). * *p* < 0.05; unpaired *t*-test.

To delineate the molecular pathways employed by Tat-SP4 for cell entry, we first synthesized Rhod-SP4, which is a stapled peptide with the same Beclin 1-targeting segment as Tat-SP4, but the N-terminal Tat sequence was replaced by rhodamine as the fluorescence dye to monitor cellular uptake by imaging studies. Flow cytometry analysis revealed that cellular uptake of Rhod-SP4 was temperature-dependent, with maximal uptake at 37 °C and markedly reduced uptake at 4 °C (Figure 5A,B). This result suggests that the internalization of Tat-SP4 is energy-dependent rather than simple diffusion.

The molecular process for Rhod-SP4 cell entry was further investigated using inhibitors of known translocation pathways (Figure 5C,D). Heparin, an inhibitor of charge-based glycosaminoglycan-mediated translocation process, did not affect the cellular uptake of Rhod-SP4. A similar result was obtained for methyl-beta cyclodextrin (MβCD), an inhibitor for clathrin- or caveolae-mediated endocytosis. In contrast, amiloride, an inhibitor of clathrin-independent macropinocytosis, significantly reduced cellular uptake of Rhod-SP4. Overall, these results suggest that Rhod-SP4 utilizes energy-dependent macropinocytosis for cell entry.

We further investigated whether the inhibitors of known translocation pathways could rescue Tat-SP4-induced cell death. Our data shows that both heparin and amiloride readily rescued cell death induced by Tat-SP4 in a dose-dependent manner (Figure 5E,F). In comparison, MβCD showed no such effect (Figure 5G). While heparin did not affect the cellular uptake of Rhod-SP4, its inhibitory effect on Tat-SP4 is likely due to the Tat sequence. As a highly negatively charged molecule, heparin may have non-specific binding to the positively charged Tat sequence, thus hindering the cell entry of Tat-SP4. Overall, our studies suggest that Tat-SP4 gains cell entry through energy-dependent and charge-mediated micropinocytosis.

### 3.6. Ca^2+^ Influx Can Serve as a Surrogate Signal to Track Macropinocytosis-Mediated Cell Entry of Tat-SP4

Direct and real-time monitoring of macropinocytosis-mediated cell entry by Tat-SP4 is not feasible because this peptide is not labeled with a trackable biomarker. As a fluorophore-labeled surrogate, Rho-SP4 can be monitored by fluorescent imaging, but the rhodamine moiety differs significantly from the Tat sequence, so its cellular uptake pathway may differ from that for Tat-SP4. As macropinocytosis is a non-selective engulfment process, we reasoned that extracellular fluids and solutes might be internalized together with Tat-SP4. Among the many solutes to be engulfed, Ca^2+^ may serve as a good surrogate signal because it is maintained at an extremely low level of ~100 nM in the cytosol. As a result, any small influx triggered by macropinocytosis could be readily monitored by genetically encoded Ca^2+^ sensors.

**Figure 6:**
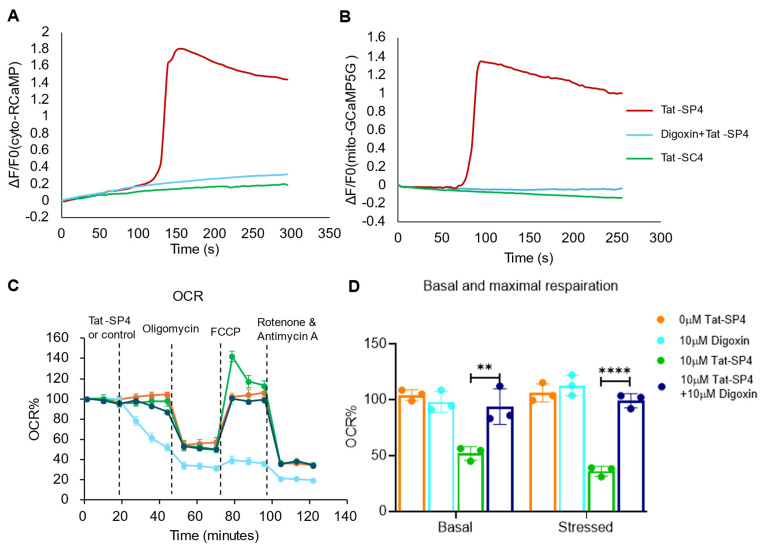
Calcium indicates Tat-SP4 uptake, and digoxin blocks entry and prevents associated cell death and OXPHOS impairment. (**A**,**B**) SHP-77 cells were transfected with cyto-RCaMP1h (**A**) or mito-GCaAMP5G (**B**) for 36 h. Treatment with 10 µM Tat-SP4 in SHP-77 cells led to an increase in cytosolic (**A**) and mitochondrial (**B**) Ca^2+^ levels (red curve). SHP-77 cells were pretreated with 10µM digoxin for 1 h, followed by the stimulation of Tat-SP4. Digoxin can inhibit the increase in cytosolic (**A**) and mitochondrial (**B**) Ca^2+^ levels (blue curve. Tat-SC4 did not disrupt the intracellular Ca^2+^ homeostasis (green curve). Each curve presents the real-time Ca^2+^ dynamics under indicated stimulations of a single cell. F0 refers to the fluorescence intensity at the start of measurement and ΔF = Ft–F0, where Ft refers to the fluorescence intensity at time t. (**C**) SHP-77 cells were pretreated with digoxin, followed by Tat-SP4 or vehicle (control), 1 μM oligomycin, 1 μM FCCP, and 0.5 μM rotenone and antimycin A sequentially. (**D**) Quantification of basal maximum respiration changes after Tat-SP4 treatment and in (**C**), ** *p* < 0.01, **** *p* < 0.0001.

To test whether cell entry by Tat-SP4 would trigger a measurable influx of extracellular Ca^2+^ into the cytosol, we transfected SHP-77 cells with two fluorescent sensors, including cyto-RCaMP to track Ca^2+^ in the cytosol and mito-GCaMP5G to track Ca^2+^ in the mitochondria, as these organelles are known to be Ca^2+^ storage sites. Our data shows that Tat-SP4 triggered increases in the fluorescence intensity for cyto-RCaMP and mito-GCaMP5G in SHP-77 cells (Figure 6A,B). In comparison, the control peptide Tat-SC4 did not induce notable changes in the Ca^2+^ signal of cyto-RCaMP or mito-GCaMP5G (Figure 6A,B). These findings suggest that the macropinocytosis utilized by Tat-SP4 for cell entry triggered a strong influx of extracellular Ca^2+^ into the cytosol, a portion of which was taken up and stored in the mitochondria. Thus, Ca^2+^ influx can serve as a direct surrogate signal to monitor entry of Tat-SP4 in real time. Additionally, these results also suggest that the cationic Tat sequence alone is not sufficient to trigger macropinocytosis, as Tat-SC4 failed to induce Ca^2+^ influx. Instead, both the N-terminal Tat moiety and the C-terminal Beclin 1-targeting segment are required for efficient cell entry of Tat-SP4 through charge-mediated macropinocytosis.

A previous study showed that digoxin could inhibit macropinocytosis by mediating the HIF signaling pathway [27]. We reasoned that, with such an inhibitory effect, digoxin would reduce the Ca^2+^ influx triggered by macropinocytosis of Tat-SP4. Indeed, our data shows that pretreatment of SHP-77 cells with digoxin completely abolished the Ca^2+^ influx triggered by Tat-SP4 as measured by cyto-RCaMP and mito-GCaMP5G (Figure 6A,B). Additionally, digoxin pretreatment also mitigated the impact of Tat-SP4 on mitochondrial function as the basal and maximum OCR levels were comparable to those for untreated controls (Figure 6C,D). Collectively, these findings further validate macropinocytosis as the major pathway for cellular uptake of Tat-SP4, which can be tracked by Ca^2+^ influx and inhibited by digoxin.

### 3.7. Tat-SP4 Exerts Potent Anti-Tumor Effect in SHP-77 Xenograft Model

**Figure 7:**
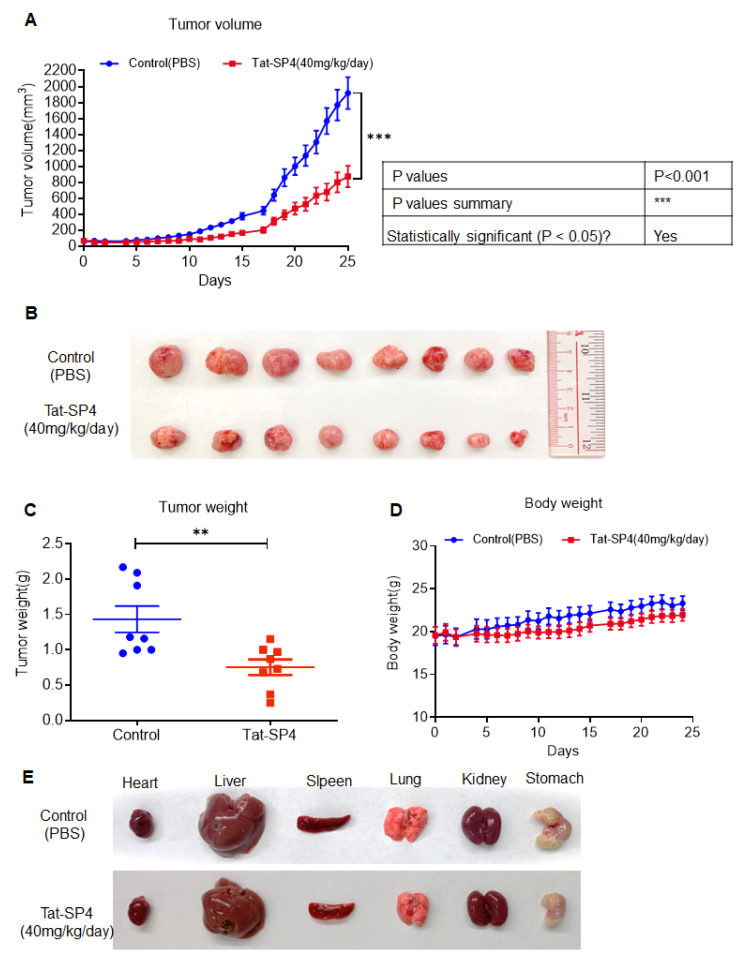
The anti-proliferative efficacy of Tat-SP4 in the SHP-77 xenograft model. (**A**) SHP-77 cells were subcutaneously injected into nude mice at a dose of 5 × 10^6^. The administration of 40 mg/kg Tat-SP4 (treatment) or PBS (control) was started when the tumor volume reached about 60 mm^3^. The tumor volume was recorded every other day. The tumor volume was defined as width^2^ × length/2. Data represents mean ± SEM, *n* = 8; *** *p* < 0.001; Two-way ANOVA. (**B**) Image of tumors at the end of the time point from the control and Tat-SP4 treatment groups. (**C**) Tumor weight on Day 25. Data represents mean ± SEM, *n* = 8; ** *p* < 0.01; unpaired *t*-test. (**D**) The changes in the mice body weight during treatment for each group. Two-way ANOVA. (**E**) Representative images of major organs (heart, liver, spleen, lung, kidney, and stomach) collected from mice after treatment.

The in vivo anti-proliferative efficacy of Tat-SP4 was investigated using the SHP-77 xenograft model. SHP-77 cells (5 × 10^6^) were implanted subcutaneously into 5-week-old male nude mice. Tat-SP4 administration began when the tumor volume reached 60 mm^3^, with a dosage of 40 mg/kg/day via intraperitoneal (i.p.) injection. Mice in the control group received PBS as a placebo. Tat-SP4 significantly inhibited tumor growth compared to PBS (Figure 7A–C). During the treatment period, no abnormal changes were observed in body weight or the gross morphology of major organs (heart, liver, spleen, lung, kidney, and stomach) (Figure 7D,E). In summary, this in vivo animal experiment demonstrated that Tat-SP4 exhibits significant anti-proliferative efficacy in the SHP-77 cell-driven xenograft model.

## 4. Discussion

Therapeutic targeting of autophagy in cancer has remained a complex but promising challenge, as autophagy modulation can either suppress or promote tumor growth in a context- and disease-dependent manner [9]. Autophagy modulators such as chloroquine and rapamycin showed anti-proliferative effects in preclinical models, but their clinical efficacy in patients was limited due to inconsistent responses, adaptive resistance, and off-target effects [9,28,29]. These limitations underscore the need for more precise and potent autophagy modulators capable of selectively disrupting cancer cell survival without triggering adaptive resistance mechanisms.

We have previously developed and characterized a series of autophagy-targeting stapled peptides that bind to the coiled coil domain of Beclin 1 to promote autophagic activity [14]. This study, together with our previous reports, has confirmed that these designed peptides, with the lead candidate Tat-SP4 as a representative, readily trigger autosis, an autophagy-dependent necrotic cell death program with distinct molecular features, including mitochondrial dysfunction [15]. This autosis-centric activity enables Tat-SP4 to overcome the dual-natured limitation of autophagy in tumorigenesis and exert a broad anti-proliferative effect in many cancer types.

For peptide-based therapeutic modalities like Tat-SP4 that must engage intracellular targets, the cell membrane is a severely restrictive barrier, as they cannot cross it by passive diffusion [30]. Instead, these large-size peptides employ one or several endocytic pathways to facilitate cell entry. However, the interplay between peptide physicochemical properties and the suitable endocytic trafficking pathways, which is critical for uptake efficiency and therapeutic potency, remains understudied.

Here, our study has identified macropinocytosis as the major pathway to facilitate cell entry of Tat-SP4. Using cell death rescue assays to screen a series of inhibitors that block specific endocytic pathways, we have confirmed that both the N-terminal cationic Tat moiety and the C-terminal Beclin 1-targeting segment are required for efficient cell entry of Tat-SP4 through charge-mediated macropinocytosis. Additionally, we demonstrated that the influx of extracellular Ca^2+^ is an effective surrogate signal to monitor the macropinocytosis of Tat-SP4 in real time. This assay would enable the study of macropinocytosis-mediated cell entry for other therapeutic modalities.

Overall, our study has confirmed the anti-proliferative effect of Beclin 1-targeting stapled peptides in SCLC and has elucidated two key mechanistic insights, including their distinct autosis-centric activity and macropinocytosis-mediated cellular uptake. Notably, cancer cells are known to exhibit hyperactive macropinocytosis as a means to scavenge extracellular nutrients and adapt to metabolic stress [31]. By exploiting this vulnerability, our designed peptides represent a promising therapeutic strategy for autophagy-dysregulated and macropinocytosis-hyperactive cancer types, including SCLC.

## Data Availability

The original contributions presented in this study are included in the article/supplementary material. Additional data related to this paper are available on request from the corresponding author.

## Supplementary Materials

The following supporting information can be downloaded at: https://www.mdpi.com/article/10.3390/pharmaceutics17121521/s1, Figure S1. Tat-SC4 exerted no significant effect autophagy in SCLC cell lines; Figure S2. Anti-proliferative efficacy of Tat-SC4 in SCLC cell lines.

## Abbreviations

SCLC: small-cell lung cancer; ∆ψm: mitochondrial membrane potential; OXPHOS: oxidative phosphorylation; ES: extensive-stage; LS: limited-stage; ICIs: immune checkpoint inhibitors; TNBC: triple negative breast cancer; PDAC: pancreatic ductal adenocarcinoma; HCC: hepato-cellular carcinoma; NSCLC: non-small-cell lung cancer; PI: propidium iodide; ETC: electron transport chain; OCR: oxygen consumption rate; CPPs: cell-penetrating peptides; MβCD: methyl-beta cyclodextrin.
