# Supplementary material for: Autophagy-Targeting Stapled Peptide Utilizes Macropinocytosis for Cell Entry to Potentiate Anti-Proliferative Autosis in Small-Cell Lung Cancer
Source: Pharmaceutics. 2025 Nov 26;17(12):1521. doi: 10.3390/pharmaceutics17121521 (PMC12736451; doi:10.3390/pharmaceutics17121521)
Supplement: Supplementary file 1 [file pharmaceutics-17-01521-s001.zip › pharmaceutics-3887959-supplementary.pdf]

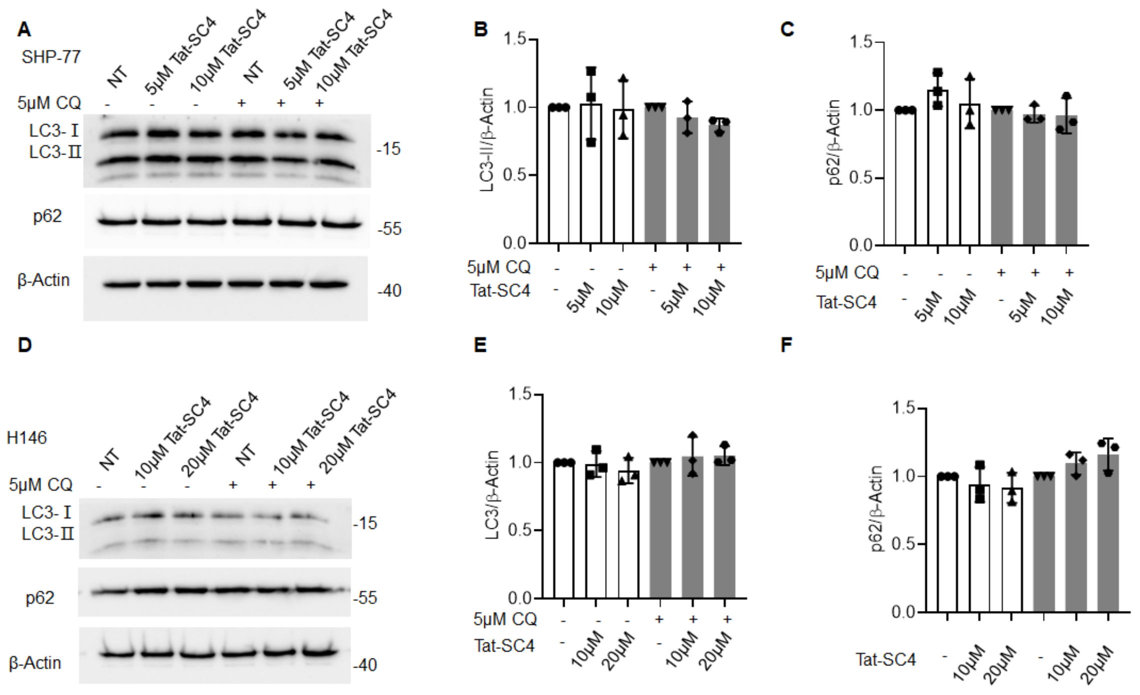

Supplementary Figure S1. Tat-SC4 exerted no significant effect autophagy in SCLC cell lines. (A) Western blot to assess the p62 level and LC3 lipidation profile in SHP-77 cells after treatment with 5μM and 10 μM of Tat-SC4 for 3h, in the presence or absence of CQ. (B) Quantification of LC3 lipidation profiles. (C) Quantification of p62 levels from the Western blot data. (D) Similar western blots as (A), but cells are H146. (E&F) Quantification of LC3 and p62 levels from data in (E). The levels of LC3-II or p62 were normalized to the β-Actin level. Data are presented as mean ± SEM (n = 3), unpaired t-test.

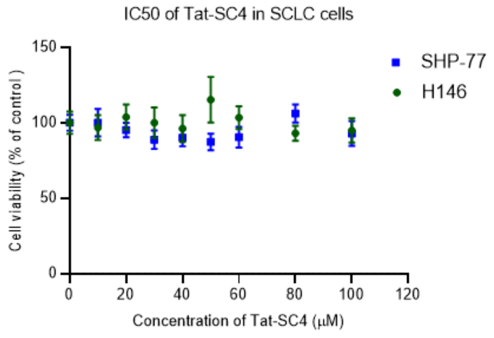

Supplementary Figure S2. Anti-proliferative efficacy of Tat-SC4 in SCLC cell lines. Trypan blue exclusion assay to assess the cytotoxicity IC50 of Tat-SPC in SHP-77 and H146 cell lines. Cells were treated with Tat-SC4 at a series of concentrations for 24 hours. The number of viable cells was determined manually using a hemocytometer and the trypan blue dye exclusion method.
